# Molecular Mechanisms Underlying the Pathogenicity of *Pseudomonas aeruginosa*

**DOI:** 10.3390/medicina62030462

**Published:** 2026-02-28

**Authors:** Angelika Krūmiņa, Aigars Reinis, Agneta Jeske, Indra Zeltiņa, Ludmila Vīksna

**Affiliations:** 1 Department of Infectology, Riga Stradins University, 1007 Riga, Latvia; 2 Department of Biology and Microbiology, Riga Stradins University, 1007 Riga, Latvia; aigars.reinis@rsu.lv; 3 Pauls Stradins Clinical University Hospital, 1002 Riga, Latvia; 4 Faculty of Medicine, Riga Stradins University, 1007 Riga, Latvia; agnetajeskee@gmail.com; 5 Riga East Clinical University Hospital, 1079 Riga, Latvia

**Keywords:** *Pseudomonas aeruginosa*, chronic infection, antibiotic resistance, virulence factors, quorum sensing, biofilm

## Abstract

*Background and Objectives*: *Pseudomonas aeruginosa* is a versatile, opportunistic pathogen responsible for a wide spectrum of infections, particularly in immunocompromised patients and those with disrupted epithelial barriers and chronic respiratory conditions. Its clinical significance is amplified by intrinsic and acquired antibiotic resistance, contributing to high mortality rates and treatment challenges. The bacterium’s pathogenic success stems from a multifaceted repertoire of virulence factors, including adhesins, pili, fimbriae, flagella, exopolysaccharides, biofilm-associated proteins, secreted toxins, proteases, lipases, phospholipases, rhamnolipids and redox-active metabolites. These factors are tightly regulated through complex networks, such as quorum sensing and c-di-GMP signaling, enabling dynamic adaptation to host environments and modulation of acute and chronic infection phenotypes. Biofilm formation and nutrient acquisition strategies further support survival in resource-limited conditions and protect against immune clearance and antibiotic pressure. Antibiotic resistance in *P. aeruginosa* limits therapeutic options. In addition, it may indirectly enhance virulence through modulation of stress responses and quorum sensing. *P. aeruginosa’s* pathogenicity emerges from the synergy between traditional virulence determinants and adaptive survival strategies, supporting long-term persistence, chronic infection, and resistance to host immunity and therapy. *Materials and Methods*: This narrative review is based on a comprehensive analysis of recent peer-reviewed literature focusing on virulence regulation, biofilm formation, nutrient acquisition strategies, and the interplay between antibiotic resistance and pathogenicity. *Results*: The reviewed evidence indicates that virulence expression in *P. aeruginosa* is highly dynamic and context-dependent, with regulatory networks integrating environmental signals to fine-tune pathogenic responses. A consistent finding across studies is the central role of biofilm-associated adaption in promoting persistence and antimicrobial tolerance. Moreover, the interaction between resistance mechanisms and global regulatory pathways appears to enhance bacterial fitness and long-term survival within the host. *Conclusions*: A deeper understanding of these interconnected mechanisms may facilitate the development of more effective anti-virulence and therapeutic strategies.

## 1. Introduction

*Pseudomonas aeruginosa* is an aerobic, rod-shaped, Gram-negative bacterium commonly associated with infections, particularly in immunocompromised patients and patients with disrupted epithelial barriers [1,2,3]. It belongs to the ESKAPE group—comprising *Enterococcus faecium*, *Staphylococcus aureus*, *Klebsiella pneumoniae*, *Acinetobacter baumanii*, *P. aeruginosa*, and *Enterobacter* spp.—a collection of highly virulent pathogens noted for their escalating antibiotic resistance [4,5]. The acronym “ESKAPE” was first coined by Louis B. Rice in 2008 to denote a group of highly virulent, multidrug-resistant bacterial pathogens that can effectively “escape” antibiotic therapy, underscoring their clinical importance in nosocomial infections. These organisms are not only becoming more widespread but are also continually developing new resistance mechanisms, making their treatment increasingly challenging [6]. Accordingly, the World Health Organization (WHO) has classified carbapenem-resistant *P. aeruginosa* as a high-priority pathogen in its 2024 Bacterial Priority Pathogens List, emphasizing the continued need for the development of novel and effective antimicrobial therapies [7].

*P. aeruginosa* continues to pose a major burden on healthcare systems worldwide, particularly within intensive care units, where it is a leading cause of ventilator-associated pneumonia, bloodstream infections and chronic respiratory diseases [8,9,10,11]. Notably, bloodstream infections caused by *P. aeruginosa* are associated with significantly higher mortality than bacteremia due to other pathogens. In one retrospective cohort, crude mortality was 50.6% in patients with *P. aeruginosa* bacteremia compared with 38.6% in bacteremia caused by other microorganisms, with attributable morality similarly elevated [12].

These high mortality rates are compounded by the bacterium’s extensive intrinsic and acquired antibiotic resistance [13]. *P. aeruginosa* exhibits intrinsic resistance to many commonly used antibiotics, including penicillins, early-generation cephalosporins, tetracyclines, and some aminoglycosides, and can rapidly acquire additional resistance mechanisms via mutations, plasmids, and efflux pumps [14,15,16,17,18]. Consequently, multidrug-resistant strains are frequently encountered, often necessitating last-resort agents such as carbapenems or colistin [19,20]. Resistance also increases the likelihood of empirical therapy, which has been shown to independently elevate mortality: in cohort of 709 bacteremia episodes, 30-day mortality was 32.3% for multidrug-resistant (MDR) infections compared with 17.2% for non-resistant strains, highlighting how resistance both limits treatment options and worsens clinical outcomes [21].

Beyond its clinical impact, *P. aeruginosa* infections also impose a substantial economic burden on healthcare systems. Prolonged hospitalizations, frequent admissions to intensive care units, and the need for complex and often costly antimicrobial therapies significantly increase treatment expenses [22,23]. Chronic and recurrent infections—particularly among patients with cystic fibrosis (CF), chronic obstructive pulmonary disease (COPD), or those requiring mechanical ventilation—further exacerbate resource utilization and overall healthcare costs [23,24,25]. Addressing these challenges requires not only effective therapeutic strategies but also a detailed understanding of the molecular mechanisms that drive its pathogenicity and persistence.

Given its clinical, economic, and therapeutic challenges, this review primarily focuses on the molecular mechanisms underlying *P. aeruginosa* pathogenicity and the processes contributing to its antibiotic resistance, aiming to provide insights into potential therapeutic targets.

## 2. Mechanisms of Host Colonization and Damage

Historically, the first recorded observation of an infection now attributed to *P. aeruginosa* was made by Charles-Emmanuel Sédillot, a French military physician, in 1850 [26]. He noted that surgical dressings often became stained with a distinctive blue-green pigment and emitted a sweet, grape-like odor—traits now known to result from the bacterial production of pyocyanin and 2-aminoacetophenone [26]. Beyond their diagnostic value, these early observed phenotypic traits reflect fundamental aspects of *P. aeruginosa* pathogenesis. Pyocyanin contributes to host tissue damage by inducing oxidative stress, impairing epithelial integrity, and disrupting immune cell function, while volatile compounds such as 2-aminoacetophenone have been implicated in bacterial communication and host–pathogen interactions [27,28]. Taken together, these characteristics explain how *P. aeruginosa* maintains a persistent presence in host tissues and creates conditions favorable for infection.

*P. aeruginosa* is a remarkably versatile bacterium capable of thriving in a wide range of environments, from soils and freshwater to man-made environments like hospital plumbing and sinks [29,30,31]. This environmental resilience allows for frequent encounters with humans, occurring through contact with contaminated medical devices, inhalation of aerosols, or entry via cuts, wounds, or compromised mucosal barriers [32,33]. While healthy individuals often clear these transient exposures without consequence, the opportunistic nature of *P. aeruginosa* allows it to take advantage of patients with chronic or burn wounds, indwelling devices, weakened immune defenses, or respiratory conditions such as CF, which is why infections caused by *P. aeruginosa* are more commonly observed in intensive care units [8,9,10,11,34]. In these cases, the bacterium can establish persistent colonization, laying the groundwork for subsequent tissue damage and infection.

Once *P. aeruginosa* encounters a susceptible host, it employs a combination of strategies to establish itself and persist. The bacterium first attaches to epithelial surfaces, damaged tissues, or medical devices using pili, fimbriae, and other adhesins [35]. Following adhesion, the bacterium can form complex biofilms—dense communities encased in an extracellular polymeric matrix—that protect it from host immune responses and environmental stresses [36]. Within these biofilms, *P. aeruginosa* can communicate and coordinate its behavior, adjusting metabolism and virulence in response to population density and local conditions [36]. Collectively, these mechanisms underpin immune evasion and persistent colonization, ultimately facilitating infection [35,36].

*P. aeruginosa* infections often begin quietly with few or nonspecific early symptoms such as mild inflammation, slight discomfort, or delayed healing, making detection challenging [37]. Over time, *P. aeruginosa* can establish persistent colonies in tissues that are difficult for the immune system to clear [38]. Commonly affected sites include the lungs, urinary tract, burn wounds and areas surrounding implanted devices, where the bacterium can form biofilms and evade host defenses [34]. As colonization progresses, patients may experience more pronounced clinical signs including fever, increased respiratory distress, purulent discharge, or worsening of chronic wounds [39]. Importantly, the marked heterogeneity among *P. aeruginosa* strains contributes to distinct clinical manifestations, which will be addressed later in this review.

Although early symptoms may be subtle or nonspecific, the underlying colonization by *P. aeruginosa* involves complex strategies that allow it to persist, evade host defenses, and adapt to local conditions. This sets the stage for chronic infection and highlights the importance of understanding the molecular mechanisms that enable the bacterium to maintain a foothold within the host.

## 3. Virulence Mechanisms of *P. aeruginosa*

The pathogenic success of *P. aeruginosa* is largely driven by its extensive repertoire of virulence factors, which enable the bacterium to colonize host tissues, evade immune defenses, and cause direct tissue damage [40]. Unlike obligate pathogens, *P. aeruginosa* relies on a flexible and highly regulated set of factors whose expression is tightly controlled in response to environmental conditions and host-derived signals [40]. This adaptability allows the bacterium to switch between acute and chronic infection modes, tailoring its virulence strategies to the specific niche it occupies within the host [41].

Consequently, not all *P. aeruginosa* strains exhibit the same virulence profile and significant differences in virulence factor composition and expression can be observed between environmental and clinical isolates, as well as among strains associated with acute versus chronic infections.

The *P. aeruginosa* genome is relatively large for a prokaryote and highly variable, with typical sizes ranging from approximately 5.5 to 7 Mbp and a substantial proportion of accessory DNA that differs between strains [42,43,44]. This accessory genome often carries genes associated with virulence and antibiotic resistance, contributing to genomic diversity and the bacterium’s ability to adapt to diverse environments and pathogenic niches, underlying much of its virulent potential [43,44].

The following sections will therefore focus on the major virulence factors of *P. aeruginosa*, highlighting their individual functions and contributions to pathogenesis within context of infection.

### 3.1. Adhesion & Colonization

Successful infection by *P. aeruginosa* requires effective adhesion to host tissues or abiotic surfaces, which constitutes the first critical step in colonization [45,46]. This initial attachment allows the bacterium to resist mechanical clearance, establish a stable foothold, and initiate interactions with host cells. Adhesion also facilitates biofilm formation, providing protection from immune defenses and environmental stresses, and supporting long-term persistence [45,46,47].

#### 3.1.1. Pili

During the early stages of colonization, *P. aeruginosa* relies on filamentous surface appendages to mediate adhesion to host tissues and abiotic surfaces [48,49]. Type IV pili contribute to early attachment and twitching motility via PilB-depedent extension and PilT-mediated retraction, thereby enabling efficient surface exploration and microcolony formation [48,49].

Their expression and dynamics are tightly regulated by multiple molecular systems: the cAMP-Vfr pathway activates transcription of PilA and related genes, the Pil-Chp and Wsp surface-sensing pathways trigger diguanylate cyclases (such as SadC and WspR) to increase intracellular cyclic di-GMP (C-DI-GMP) upon surface contact, and this elevated c-di-GMP then suppresses motility while promoting stable adhesion and biofilm initiation [50,51,52]. Beyond their role in motility, type IV pili also facilitate stable adhesion through interactions with host cell receptors, including asialo-GM1 glycosphingolipids on respiratory epithelial cells [53,54].

#### 3.1.2. Fimbriae

Cup fimbriae, assembled through the chaperone-usher pathway, are composed of major pilin subunits that polymerize into rigid, rod-like fibers on the bacterial surface [55,56]. These fimbriae are stabilized by specific chaperones in the periplasm and secreted through outer membrane usher proteins, ensuring proper folding and surface display [55,56]. Cup fimbriae mediate high-affinity binding to host glycoconjugates and extracellular matrix components, reinforcing attachment established by type IV pili. Their expression is tightly regulated by two-component systems and surface-sensing pathways, including Roc and Rcs/Pvr networks, which respond to surface contact and environmental cues and promote stable adhesion and early biofilm formation [57,58].

In summary, type IV pili and cup fimbriae act in a complementary manner to mediate adhesion and colonization. Type IV pili initiate surface attachment and enable twitching motility to explore and organize microcolonies, while cup fimbriae stabilize this attachment and promote early biofilm formation.

#### 3.1.3. Flagella

In *P. aeruginosa*, flagella enable swimming in liquid environments and swarming over semi-solid surfaces, the latter requiring the production of rhamnolipid biosurfactants that reduce surface tension and facilitate coordinated surface movement [59]. This motility allows the bacterium to reach and explore potential colonization sites, including epithelial tissues, wounds and medical devices [59].

Flagellar biosynthesis is hierarchically regulated. The transcription factor FleQ, together with the sigma factor RpoN (σ^54^), activates early flagellar genes (Class II genes), which encode basal body components, motor proteins and regulatory factors, including the anti-sigma factor FlgM. FlgM transiently inhibits the late sigma factor FliA (σ^28^) until assembly of the basal body and hook is completed [60,61,62]. Upon completion of these structures, FlgM is exported, allowing FliA to activate Class III and IV genes encoding hook-associated proteins, the filament cap (FliD), and flagellin (FliC), which polymerizes to form the external filament [63].

This ordered cascade ensures proper assembly and functionality of the flagellum. Flagellar gene expression is further modulated by intracellular c-di-GMP, which acts as a central switch between motile and sessile lifestyles [63]. Low c-di-GMP levels, typical of planktonic growth and acute infections, promote flagellar gene expression and motility. In contrast, elevated c-di-GMP levels, commonly associated with surface attachment and chronic infections, repress flagellar programs while favoring biofilm formation [63]. Mechanosensory feedback from flagellar rotation upon surface contact contributes to this regulatory shift by modulating the expression of genes involved in adhesion and microcolony formation [64]. The resulting flagellum enables swimming and swarming motility, facilitates direct attachment to host surfaces, and presents flagellin to host immune receptors such as TLR5, thereby integrating structural, sensory and virulence functions during colonization and infection [65].

Mutants lacking flagella exhibit impaired colonization, reduced biofilm formation, and altered virulence, highlighting the multifaceted role of flagella in both acute and chronic infections [66].

#### 3.1.4. Lectins

Lectins are carbohydrate-binding proteins that mediate specific adhesion of *P. aeruginosa* to host cells and surfaces and are predominantly associated with the outer membrane and the extracellular biofilm matrix. The main lectins, LecA (PA-IL) and LecB (PA-IIL), recognize distinct sugar moieties on host glycoconjugates [67,68]. LecA binds primarily galactose residues on host glycoproteins and glycolipids, while LecB targets fucose and mannose residues on cell-surface glycoproteins and extracellular matrix components [67,68]. In these interactions, the oligosaccharide ligand is part of the host cell, whereas the carbohydrate-binding site resides in the bacterial lectin oligomer [67,68].

Structurally, LecA forms a tetramer, and LecB a tetrameric or dimeric assembly, creating multivalent binding sites [67]. This multivalency strengthens adhesion by allowing simultaneous interactions with multiple glycan ligands on the host surface [67]. In biofilms, lectins bind to exopolysaccharides (EPS) such as Psl and Pel, stabilizing the extracellular polymeric matrix and enhancing bacterial aggregation and biofilm integrity [69].

Lectin expression is primarily regulated by quorum sensing systems, especially, through the RhlR/C4-HSL pathway, which directly influences the transcription of lecA and lecB [70]. In addition, the alternative sigma factor RpoS is required for full lectin synthesis, linking lectin expression to the general stress response [70]. Although other global regulators and environmental signals likely contribute to modulation of lectin expression, quorum sensing and RpoS remain the most well-characterized regulators of lectin production in this organism [70].

Lectins not only facilitate stable attachment to epithelial cells but also modulate host immune responses [71]. LecB interferes with host immunity by cross-linking glycosylated receptors on leukocytes and endothelial cells, impairing transendothelial migration and adaptive immune responses [71]. Experimental inhibition of lectin-carbohydrate interactions significantly reduces bacterial adhesion and biofilm formation, highlighting the therapeutic potential of lectin-targeted strategies [72].

#### 3.1.5. Exopolysaccharides

EPS are carbohydrate polymers secreted by *P. aeruginosa* that form the main component of the biofilm matrix. The three primary EPS—Psl, Pel, and alginate—have distinct roles [73].

Psl is a mannose/glucose/rhamnose polymer that promotes initial surface attachment, cell–cell cohesion, cell-surface interaction, and microcolony formation. It also helps protect bacteria from host defenses, including phagocytosis and complement attack [73,74].

Pel is a glucose-rich polymer that stabilizes cell aggregates and interacts with extracellular DNA to strengthen biofilm structure [73,75].

Alginate is an acetylated polymer of mannuronic and guluronic acids. It is overproduced in chronic infections, especially in CF lungs, forming a mucoid biofilm that protects bacteria from antibiotics and immune factors [73,76].

The production of these polysaccharides is tightly regulated. Psl expression is controlled by the alternative sigma factor RpoS and by post-transcriptional regulators such as RsmA [73,77]. Alginate and Pel biosynthesis share common sugar precursors, which are processed by the enzyme AlgC, and their relative production is modulated by regulatory proteins such as AmrZ, FleQ and the RNA-binding protein RsmA [73]. These regulators sense and respond to environmental cues such as nutrient limitation, osmotic stress, oxygen availability and host-derived signals, as well as infection stage [73]. As a result, Psl predominates during early surface attachment and initial biofilm formation, Pel contributes mainly to microcolony maturation and cell–cell aggregation, and alginate dominates in chronic infections [73].

#### 3.1.6. Biofilm-Associated Adhesins

Other adhesins of *P. aeruginosa* complement the primary adhesion factors such as pili, fimbriae, and lectins by reinforcing bacterial attachment and biofilm integrity.

The biofilm-associated protein CdrA is secreted through a two-partner secretion system encoded by the cdrAB operon and functions as a key adhesin within the biofilm matrix [78]. Once exported to the cell surface, CdrA binds specifically to the Psl exopolysaccharide, forming cross-links between bacterial cells and the extracellular matrix [78,79]. This interaction enhances cell–cell cohesion, promotes microcolony stability, and contributes to the structural integrity of mature biofilms [79]. Disruption of CdrA-Psl interactions leads to impaired aggregation and mechanically weaker biofilms, highlighting the importance of CdrA as a matrix-stabilizing adhesin during colonization and biofilm development [78].

### 3.2. Tissue Damage & Secreted Factors

Once stable attachment and colonization are established, *P. aeruginosa* deploys a diverse set of secreted virulence factors that damage host tissues and promote bacterial dissemination. The production of these factors marks a functional transition from surface-associated colonization to active tissue invasion and progression toward invasive disease [80].

#### 3.2.1. Type III Secretion System

The Type III Secretion system (T3SS) of *P. aeruginosa* is a needle-like apparatus that delivers effect proteins directly into host cells [80]. It forms a translocon pore in the host membrane, allowing effectors to bypass the extracellular environment and act intracellularly [81]. This system is crucial for acute infections because it allows the bacterium to disrupt host cell processes and evade immune defenses. Only four effectors have been identified—ExoS, ExoT, ExoU, and ExoY [80].

ExoS is a bifunctional protein with GTPase-activating (GAP) and ADP-ribosyltransferase (ADPRT) domains [82,83]. The ADPRT domain transfers ADP-ribose to host proteins such as Ras, Rab and Rho GTPases, disrupting signaling pathways that control the actin cytoskeleton [82]. The GAP domain inactivates Rho, Rac1 and CDC42, leading to cytoskeletal collapse [82,84,85]. Through these combined activities, ExoS effectively inhibits phagocytic uptake by immune cells [82]. ExoS can also trigger apoptosis through these pathways [82,86].

ExoT shares structural similarity with ExoS and contains ADPRT and GAP domains [82,83,87]. Its ADPRT activity targets Crk adaptor proteins and phosphoglycerate kinase 1 (PGK1), disrupting focal adhesion formation and interfering with host cell signaling [88]. The GAP domain inactivates RhoA, Rac1 and Cdc42, leading to cytoskeletal alterations and changes in cell morphology, and inhibition of actin-dependent phagocytic uptake by immune cells [82,89]. Interestingly, ExoT can induce apoptosis through two distinct mechanisms: ADPRT-mediated anoikis apoptosis occurs rapidly, within 5.5 ± 1.3 h, whereas GAP-mediated mitochondrial apoptosis proceeds more slowly, within 16.2 ± 1.3 h in intoxicated cells [90]. ExoT is also the only T3SS effector expressed in all T3SS-positive *P. aeruginosa* clinical strains, highlighting its fundamental role in the pathogenesis of this bacterium [91].

ExoU is a potent phospholipase A_2_, activated by host cofactors ubiquitin and phosphatidylinositol 4,5-bisphosphate (PI (4,5) P2) [82,92]. Once activated, it hydrolyzes phospholipids in the host plasma membrane, causing rapid cell lysis [93]. Strains expressing ExoU are frequently associated with poor clinical outcomes. Approximately 28–48% of clinical *P. aeruginosa* isolates carry the *exoU* gene, and ExoU-positive strains are predominantly identified in severe infections such as pneumonia and other critical hospital-acquired conditions, where their cytotoxic activity contributes to worse patient outcomes [94,95]. ExoU-induced disruption of the endothelial barrier can trigger excessive inflammatory responses, which in animal models of pneumonia has been shown to lead to acute respiratory distress syndrome (ARDS) [96]. In addition, ExoU-positive isolates have been reported in multiple studies to exhibit higher rates of multidrug resistance, especially with fluoroquinolone resistance, which complicates treatment and contributes to their clinical impact [94].

ExoY functions as a host-activated nucleotidyl cyclase, exhibiting both adenylyl and guanylyl cyclase activities [82,97]. After delivery into host cells, ExoY becomes enzymatically active upon binding to filamentous actin (F-actin) [97]. This interaction triggers the synthesis of cyclic nucleotides, predominantly cAMP and cGMP, leading to abnormal accumulation of intracellular second messengers [97]. The elevation of cyclic nucleotide levels disrupts host cell signaling pathways that regulate cytoskeletal organization and barrier integrity [97]. As a result, ExoY induces disassembly of the actin cytoskeleton and impairs endothelial and epithelial junctions [97]. These changes increase vascular permeability, promote tissue edema, compromise endothelial repair following injury, and interfere with phagocytic uptake by immune cells [97,98]. In clinical and experimental studies, ExoY expression has been associated with increased vascular permeability, organ dysfunction, and more severe disease manifestations during acute *P. aeruginosa* infections [97,99].

T3SS expression is tightly controlled by the transcriptional activator ExsA, which is released upon host cell contact through a partner-switching mechanism involving ExsC, ExsD and ExsE [100]. In the absence of host interaction, ExsE sequesters ExsC, preventing ExsA activation. Upon host cell contact, ExsE is secreted, allowing ExsC to bind ExsD and thereby liberating ExsA to activate transcription of T3SS structural genes and effectors [100,101]. T3SS expression can also be modulated by environmental cues such as extracellular calcium levels and intracellular second messengers, linking its activation to both host interaction and broader bacterial regulatory networks [101]. Functionally, ExsA mutants exhibit severely reduced cytotoxicity toward host cells and are more susceptible to phagocytosis, likely due to the ExsA-dependent transcription of impA and other virulence genes [102].

#### 3.2.2. Secreted Proteases

*P. aeruginosa* produces a well-defined set of extracellular proteases that contribute significantly to tissue destruction, bacterial dissemination, and modulation of host defense mechanisms during infection [40,103]. The major secreted proteases implicated in virulence include elastase B (LasB) and elastase A (LasA), alkaline protease (AprP), and protease IV (PIV). Together they form a coordinated proteolytic arsenal that enables *P. aeruginosa* to degrade structural components of the extracellular matrix, disrupt epithelial and endothelial barriers, and selectively inactivate immune effector molecules, thereby facilitating both acute tissue injury and sustained infection [40,103].

Elastase B (LasB) is a zinc- and calcium-dependent metalloprotease of the thermolysin-type M4 peptidase family that is a major virulence factor of *P. aeruginosa* [104]. At the molecular level, elastase B cleaves key extracellular matrix (ECM) proteins, including elastin, collagen, laminin and fibronectin, and fibrin, resulting in weakening the tissue architecture and loss of integrity [104,105]. In addition to structural substrates, elastase B targets host immune components such as complement factor C1q and C3 and pulmonary surfactant proteins (SP-A, SP-D), impairing opsonization, complement-mediated killing, and epithelial defense mechanisms [103,104,106]. Production of elastase B is regulated by quorum sensing, primarily through the LasR-LasI system, linking its expression to bacterial population density and coordinated virulence [107]. Importantly, in vivo studies in murine models of acute lung injury have shown that purified elastase B alone can induce diffuse alveolar damage, hemorrhage, and hyaline membrane formation, confirming its potent tissue-destructive and toxic effects during infection [108].

Elastase A (LasA) is an extracellular serine protease secreted by *P. aeruginosa* [40,109]. Unlike LasB, LasA does not extensively degrade elastin on its own [109]. Instead, LasA introduces initial cleavages to elastin fibers [109]. These structural disruptions make elastin a more accessible substrate for other proteases, particularly LasB, thereby enhancing overall tissue degradation [109]. In addition to its activity on host proteins, LasA exhibits staphylolytic activity by cleaving the pentaglycine cross-bridges in the peptidoglycan of *Staphylococcus aureus* [110]. This activity reflects a narrow and specific substrate preference rather than broad proteolysis [110]. LasA is secreted as an inactive proenzyme and becomes activated extracellularly through proteolytic processing by other secreted proteases [111]. Although LasA alone has limited destructive capacity, its synergistic function within the protease network of *P. aeruginosa* contributes to efficient breakdown of host barriers during infection [112].

Alkaline protease (AprA) is a secreted zinc-dependent metalloprotease of *P. aeruginosa* that belongs to M10 peptidase family [113,114]. It functions primarily as an immune-modulating virulence factor [115]. Rather than directly degrading structural extracellular matrix components, AprA selectively targets host immune proteins, with a major impact on the complement system [115]. AprA has been shown to degrade C1q, the recognition molecule of the classical pathway, as well as C1s and C2, thereby inhibiting activation of both the classical and lectin pathways [115,116]. This activity prevents effective C3b deposition and reduces C5a generation, resulting in significantly impaired complement-mediated bacterial killing, neutrophil recruitment, and phagocytosis [115]. AprA has also been reported to disrupt neutrophil extracellular traps (NETs) and degrade immune-stimulatory molecules, further promoting bacterial survival and persistence during infection [117]. AprA has been shown to contribute to pyocyanin production, with aprA-complemented strains synthesizing higher levels of this pigment than aprA mutant strains, indicating a role for AprA-mediated proteolysis in pyocyanin biosynthesis [118].

Expression of alkaline protease is tightly regulated and linked to bacterial population density and environmental conditions [119]. The *aprA* gene is part of the *apr* operon and is positively regulated by quorum sensing, primarily through the LasR-LasI system, with additional contribution from the RhlI-RhlR system [120]. Alkaline protease is exported via a type I secretion system that requires three membrane-associated proteins—AprD, AprE, AprF [121]. The genes encoding these components are located immediately upstream of the *arpA* structural gene, forming a dedicated operon that facilitates direct translocation of aprA into the extracellular environment [121].

Protease IV (PIV) is a secreted serine protease belonging to the S1 peptidase family [122]. It functions as a virulence factor by modulating host immune responses and contributing to tissue damage. PIV degrades multiple host immune proteins and components involved in innate defense, including surfactant proteins and the cytokine IL-22, thereby interfering with immune signaling and host defense mechanisms [123,124]. Unlike AprA, PIV is secreted via the type II secretion system (Xcp) where it is released as a pre-proenzyme and activated extracellularly [125]. Expression of PIV is regulated by quorum sensing, primarily via the LasR-LasI system, with further modulation by the RhlI–RhlR system and is typically induced under conditions of high cell density and specific environmental cues [125]. Functionally, PIV contributes to *P. aeruginosa* virulence in various infection models, including corneal infections, chronic wounds and pulmonary infections, by promoting bacterial survival and persistence within host tissues [123,126,127].

#### 3.2.3. Lipases and Phospholipases

*P. aeruginosa* produces a variety of extracellular enzymes that break down lipids, including lipases and phospholipases. These enzymes play a dual role in infection: they can directly damage host cell membranes, and they provide the bacterium with nutrients by releasing fatty acids and glycerol from host lipids [128]. This ability to exploit host lipids helps *P. aeruginosa* grow even when nutrients are scarce, which is often the case during infection.

Among these enzymes, lipase A (LipA) is the most abundant extracellular lipase produced by *P. aeruginosa*. It hydrolyzes triglycerides and other glycerol-based lipids, generating free fatty acids and glycerol that the bacterium can use for energy [128]. LipA belongs to the I.1 family of serine hydrolases and contains a canonical Ser-His-Asp triad, which mediates the nucleophilic attack on lipid substrates [129]. The enzyme is synthesized as an inactive precursor with an N-terminal Sec signal peptide that directs it across the inner membrane [130]. In the periplasm, folding is assisted by the co-expressed foldase LipH and chaperones such as Skp, ensuring correct conformation prior to secretion [130,131]. LipA is exported via the type II secretion system and activated extracellularly by cleavage of its propeptide, allowing it to function efficiently in the host environment [130]. Beyond nutrient acquisition, LipA contributes to tissue damage, promotes the activity of other virulence factors, and influences biofilm architecture, making infections harder to clear [130]. Its expression is tightly regulated by quorum sensing (LasR/LasI and RhlR/RhlI) and environmental signals such as nutrient availability and pH, enabling *P. aeruginosa* to optimize enzyme production during infection [132,133].

*P. aeruginosa* produces multiple phospholipases C (PLCs) with distinct substrate specificities and functional roles [134]. The hemolytic phospholipase C (PlcH) hydrolyzes phosphatidylcholine (PC) and sphingomyelin (SM), key components of eukaryotic cell membranes, which enables it to directly damage host tissues, disrupt pulmonary surfactant, and lyse red blood cells in vitro [134,135,136]. In contrast, the non-hemolytic phospholipase C (PlcN) exhibits narrower substrate specificity, primarily targeting PC and phosphatidylserine (PS), and lacks hemolytic activity [134]. While PlcN is less cytotoxic, it still contributes to nutrient acquisition and modulation of host lipid signaling. Both enzymes are synthesized as precursors with N-terminal signal peptides and are secreted via the type II secretion system, highlighting a shared export pathway despite their functional differences [137]. The distinct substrate preferences of PlcH and PlcN underlie their complementary roles in pathogenesis, with PlcH primarily driving tissue damage and immune modulation, and PlcN supporting metabolic adaptation and persistence within host tissues [137].

Other phospholipases, such as phospholipase D (PldD), are less well characterized but may contribute to host lipid modulation and bacterial survival, suggesting that multiple extracellular lipolytic enzymes act synergistically to promote virulence and persistence [138].

#### 3.2.4. Rhamnolipids

*P. aeruginosa* produces biosurfactant molecules known as rhamnolipids, glycolipids composed of one or two rhamnose sugar moieties linked to fatty acid tails [139]. The biosynthesis of rhamnolipids occurs in three enzymatic steps. First RhlA (rhamnosyltransferase chain A) converts β-hydroxy fatty acids into a dimer molecule [140,141]. Next, RhlB (rhamnosyltransferase chain B) attaches this dimer to dTDP-L-rhamnose to form mono-rhamnolipids [142]. Finally, RhlC (rhamnosyltransferase II) adds a second dTDP-L-rhamnose to produce di-rhamnolipids, the main rhamnolipid species secreted by *P. aeruginosa* [139,140,143]. Rhamnolipid biosynthesis is tightly controlled by the rhl and rhlAB quorum sensing system, which controls the expression of the *rhlAB* operon encoding a rhamnosyltransferase enzymes [107]. This regulatory system integrates cell density signals with other circuits, including Las and Pqs, to coordinate rhamnolipid production according to population status and environmental conditions [144].

Functionally, rhamnolipids reduce surface and interfacial tension, facilitating swarming motility and dispersion of bacterial communities, and they are critical for the structural development and maintenance of biofilms [139,145]. By altering cell–cell and cell-surface interactions, rhamnolipids promote detachment of cells from mature biofilms and can mediate biofilm architecture remodeling, which contributes to chronic infection persistence and antibiotic tolerance [139,145].

Rhamnolipids also interact directly with host cells and immune effectors. Their amphipathic nature allows them to insert into eukaryotic membranes, leading to membrane destabilization and cytotoxicity at high concentrations, as secreted rhamnolipids can form micellar structures that rapidly rupture the plasma membrane and damage intracellular organelles in macrophages [139]. Moreover, rhamnolipids have been shown to modulate host immune responses by influencing neutrophil chemotaxis, altering cytokine production, and interfering with phagocytosis, thereby contributing to immune evasion and prolonged infection [146,147].

Finally, rhamnolipid production affects virulence factor delivery and interacts with other secreted products such as elastases and proteases, forming a coordinated virulence network that enhances tissue destruction and pathogen spread. Their relatively conserved biosynthetic pathway and strong regulatory control underscore their importance in *P. aeruginosa* pathogenesis [109].

#### 3.2.5. Redox-Active Metabolites

*P. aeruginosa* produces small molecules called redox-active metabolites, which play a key role in infection. These compounds, such as phenazines, can transfer electrons and create reactive oxygen species (ROS) [148]. Ros can damage host cells and tissues by disrupting membranes and organelles. Redox-active metabolites also interfere with immune cells, reducing their ability to fight infection. In addition, these molecules help the bacteria survive in low-oxygen environments and within biofilms. Overall, redox-active metabolites act both as toxins and as tools for bacterial survival, making them central to *P. aeruginosa* virulence [148].

Pyocyanin is a blue-green pigment and the most studied phenazine produced by *P. aeruginosa* [148]. It is redox-active molecule that cycles between oxidized and reduced states, accepting electrons from cellular donors such as NADH and NADPH and transferring them to molecular oxygen [149]. This redox cycling generates ROS, including superoxide anion (O_2_^−^), hydrogen peroxide (H_2_O_2_), and hydroxyl radicals (•OH) [148,149]. These ROS cause lipid peroxidation in host cell membranes, oxidize protein thiol groups, and inactive iron-sulfur cluster-containing enzymes, disrupting metabolism [150]. Pyocyanin also targets mitochondria, collapsing the membrane potential, inhibiting the electron transport chain, and depleting ATP. In immune cells, ROS produced by pyocyanin reduce NADPH and glutathione levels, impairing neutrophil oxidative burst and macrophage phagocytosis [149,150]. Calcium signaling is disrupted, triggering apoptosis or necrosis depending on dose and exposure [149]. In epithelial and endothelial tissues, these effects compromise barrier integrity, facilitating bacterial invasion [151]. Within biofilms, pyocyanin functions as an extracellular electron shuttle, maintaining redox balance under hypoxic conditions [152]. Its production is tightly regulated by quorum sensing systems. LasI, RhlR/RhlI, and PqsR/PQS, linking synthesis to cell density and environmental signals, and coordinating virulence factor expression during infection [153].

A key study revealed a novel molecular mechanism by which pyocyanin contributes to biofilm stability [154]. Pyocyanin can directly bind extracellular DNA (eDNA), a major component of the biofilm matrix. This interaction enhances biofilm viscoelasticity and mechanical stability, making bacterial communities more resilient [154]. Using pyocyanin-deficient mutants, the study showed that loss of pyocyanin reduced matrix density and elasticity, whereas exogenous pyocyanin restored these properties. Additionally, pyocyanin-eDNA complexes were found to influence sputum physical characteristics, which is particularly relevant in chronic lung infections such as cystic fibrosis [154]. These findings indicate that pyocyanin functions not only as an ROS-generating cytotoxin but also as a molecular scaffold, organizing biofilm structure and promoting persistence and resistance to host defenses and antibiotics [154].

### 3.3. Host Immune Evasion & Interference

*P. aeruginosa* has evolved a sophisticated arsenal to evade and manipulate the host immune system [40]. It produces exopolysaccharides such as alginate, Psl and Pel, which assemble into a protective biofilm matrix [73]. This barrier impedes immune cell infiltration, reduces complement activation, and limits opsonization, allowing bacterial communities to persist within host tissues [73]. Biofilms also trap neutrophils and macrophages at their periphery, resulting in ineffective clearance and sustained inflammation [73].

A key factor in immune evasion is the bacterium’s lipopolysaccharide (LPS). LPS consists of three parts: lipid A, a core oligosaccharide, and the O-antigen polysaccharide [155]. *P. aeruginosa* can modify lipid A using enzymes such as PagL and LpxO [156]. These modifications change the number and arrangement of fatty acid chains and phosphate groups, which makes LPS less recognizable by Toll-like receptor—4 (TLR4). As a result, NF-κB signaling is reduced and fewer pro-inflammatory cytokines are produced, allowing the bacteria to establish infection before the immune system fully responds [156].

The O-antigen forms long sugar chains that act like a shield [157]. It physically blocks the binding of complement component C3b and prevents the formation of the membrane attack complex, protecting bacteria from complement-mediated killing [157]. Some strains can even recruit host complement regulatory proteins to the LPS surface, providing an additional layer of defense [157].

Finally, *P. aeruginosa* exhibits variability in O-antigen. Length and composition, generating heterogeneous populations that are harder for antibodies to recognize and adapt to different host tissues [158].

Within this LPS-protected environment, *P. aeruginosa* secretes extracellular proteases such as LasA, LasB, AprA and protease IV, which degrade antibodies, cytokines, surfactant proteins, and complement components [103]. These enzymes weaken immune signaling and reduce phagocyte effectiveness, further weakening the host response [103]. To act even more directly, *P. aeruginosa* uses the T3SS to inject effectors such as ExoS, Exot, and ExoU into host cells, disrupting cytoskeletal dynamics, blocking phagocytosis, modulating apoptosis, and interfering with intracellular signaling [80].

Inside immune cells, the bacterium resists reactive oxygen and nitrogen species by producing enzymes such as catalase, superoxide dismutase and peroxidases, which neutralize hydrogen peroxide and superoxide radicals [159]. These defenses allow *P. aeruginosa* to survive within phagosomes and avoid oxidative killing [159]. This intracellular persistence not only shields the bacteria from extracellular immune factors but also provides a reservoir from which chronic infection can be maintained, linking oxidative stress resistance to long-term survival within the host [159].

In addition to subverting innate immune defenses, *P. aeruginosa* also modulates adaptive T-cell-mediated immunity. Effective clearance of infection depends in part on Th1 and Th17 responses, which promote neutrophil recruitment and antimicrobial activity at mucosal surfaces [11,38]. However, during chronic infection this response becomes dysregulated, resulting in persistent inflammation without efficient bacterial eradication. Biofilm formation further impairs effective antigen presentation and sustains prolonged immune activation [38]. Moreover, virulence factors such as pyocyanin have been shown to modulate pulmonary immune responses and influence cytokine signaling pathways, including IL-23-associated mechanisms linked to Th17 polarization [149]. This imbalance between protective and pathological T-cell responses contributes to immune evasion, chronic persistence and progressive tissue damage.

Together, these strategies—biofilm formation, LPS-mediated masking, enzymatic degradation, direct interference with immune cells, and long-term adaptive changes—allow *P. aeruginosa* not only to evade host defenses but also to actively modulate immune functions, securing a persistent niche and driving prolonged infection and tissue pathology [40].

### 3.4. Biofilm Formation

*P. aeruginosa* biofilm formation is a cornerstone of its pathogenic success, allowing the bacterium to survive hostile host environments and resist both immune responses and antibiotic treatment [36]. Biofilms are structured communities of bacterial cells embedded in an extracellular matrix composed primarily of exopolysaccharides (alginate, Psl and Pel), along with eDNA and proteins [36]. This matrix provides both mechanical stability and protection from immune effectors. Alginate contributes to the mucoid phenotype often observed in chronic infections, particularly in cystic fibrosis airways, providing a viscous barrier that impedes phagocytosis and reduces neutrophil-mediated killing. Psl and Pel polysaccharides are essential for surface adhesion, intercellular aggregation, and matrix integrity, while eDNA reinforces the biofilm structure and stability [36].

Biofilm development is controlled by a complex regulatory network that integrates environmental signals, cell density, and stress cues [36,73]. The second messenger c-di-GMP plays a central role by promoting matrix production and biofilm maturation, while simultaneously repressing motility, allowing bacteria to shift from planktonic to sessile lifestyles [160]. Upstream, the GacS/GacA two-component system activates small RNAs, *rsmY* and *rsmZ*, which bind and sequester the RNA-binding protein RsmA, releasing repression of biofilm-associated genes, including *pel*, *psl*, and *alginate* biosynthetic operons [73]. In contrast, RetS counteracts this pathway to favor planktonic growth and acute virulence traits [73].

At the community level, quorum sensing (QS) systems—Las, Rhl, and PQS—coordinate the expression of matrix components, extracellular enzymes, and secondary metabolites, stabilizing the biofilm and linking population density to biofilm-specific virulence [73]. Additional regulators, such as FleQ, AmrZ, AlgU, AlgR, and sigma factors like RpoS, fine-tune matrix gene expression in response to environmental and stress cues. Together, these systems allow *P. aeruginosa* to dynamically adjust biofilm formation and persist under diverse host conditions [161].

Biofilm formation occurs through a well-defined, multistage process, involving initial reversible surface attachment, irreversible adhesion, microcolony formation, maturation into three-dimensional structures, and eventual dispersal of planktonic cells [36]. This dynamic lifecycle supports persistent colonization and dissemination within the host [36].

Recent time-resolved transcriptomics studies have revealed that biofilm development is temporally coordinated and shaped by host–pathogen interactions [162]. In CF airways, biofilm formation reflects a dynamic interplay between bacterial adaption and host responses, contributing to chronic infection and therapeutic failure. These insights highlight potential intervention strategies targeting matrix production, quorum sensing, and stress-response pathways to improve treatment outcomes [162].

### 3.5. Quorum Sensing: Master Regulator of P. aeruginosa Virulence

Quorum sensing (QS) functions as a central regulatory network in *P. aeruginosa*, coordinating the expression of virulence factors, biofilm formation, and metabolic adaptation. The bacterium employs interconnected QS systems, including Las (LasI/LasR), Rhl (RhlI/RhlR), PQS (Pseudomonas Quinolone Signal), and IQS, which integrate information about cell density and environmental cues [35,163].

The Las system hierarchically activates the Rhl and PQS pathways, thereby orchestrating population-level expression of extracellular proteases, toxins, siderophores, and biofilm matrix components [163]. PQS additionally links QS to stress response and secondary metabolite production, fine-tuning bacterial adaptation under hostile conditions [164]. At the molecular level, QS interacts with global regulatory networks such as Gac/Rsm, c-di-GMP signaling, and transcription factors including FleQ, RpoS, and AmrZ, ensuring that biofilm development, motility and virulence factor synthesis are tightly coordinated with environmental and metabolic states [164]. Through this hierarchical and interconnected network, QS enables *P. aeruginosa* to form mature, heterogeneous biofilms, resist host immune defenses, and persist during chronic infections [164]. The centrality of QS also makes it a promising therapeutic target, with quorum quenching strategies shown to reduce biofilm integrity and virulence factor production, potentially enhancing antibiotic efficacy and clinical outcomes [165].

QS also contributes to the transition between acute and chronic infection states [164]. During acute infections, QS activity is typically dominated by an intact and highly active Las system [164]. LasR-Lasl signaling drives the expression of numerous classical virulence factors, including elastase, alkaline protease, exotoxins, and components of the T3SS, thereby promoting rapid tissue damage, immune activation, and bacterial dissemination [164]. In this context, QS supports a planktonic, motile lifestyle optimized for invasion and exploitation of host resources [164].

In contrast, chronic infections, such as those observed in cystic fibrosis airways—are characterized by a profound reprogramming of QS networks rather than their complete inactivation [164]. According to D’Argenio et al. *lasR* mutants exhibit a loss of function in the Las quorum-sensing regulatory system, resulting in decreased expression of key quorum sensing-controlled virulence factors such as elastase and other extracellular proteases. This impairment disrupts coordinated regulation of virulence gene transcription, thereby reducing the bacterium’s ability to cause acute infection. Consequently, lasR mutants display attenuated virulence phenotypes compared to wild-type strains of *P. aeruginosa* [166]. However, downstream QS systems, particularly Rhl and the PQS pathway, often remain active or become functionally dominant [167]. This QS rewiring shifts bacterial behavior toward biofilm formation, stress tolerance, and long-term persistence. PQS signaling integrates QS with iron homeostasis, oxidative stress responses and phenazine production, reinforcing survival within the heterogeneous and nutrient-limited biofilm environment [164,165]. This regulatory plasticity underlies the remarkable ability of the pathogen to survive diverse host environments, evade therapeutic pressure, and sustain long-term infections.

### 3.6. Nutrient Acquisition

*P. aeruginosa* thrives in nutrient-limited and hostile host environments by efficiently scavenging essential metals and flexibly adjusting its metabolism [168]. Iron is a key limiting factor [168]. The bacterium produces siderophores, mainly pyoverdine (Pvd) and pyochelin (Pch), to extract iron from host proteins. Pyoverdine binds Fe^3+^ with high affinity and delivers it via the FpvA receptor [169]. Pyochelin complements pyoverdine, providing additional iron under varying conditions. Siderophore synthesis is tightly regulated by the Fur repressor, the PvdS sigma factor, and quorum sensing, linking iron acquisition to population density, virulence, and stress adaptation [169,170].

In hypoxic or anoxic biofilm zones, *P. aeruginosa* activates anaerobic respiration, often using denitrification pathways [171]. Denitrification is the stepwise reduction of nitrate (NO_3_^−^) to nitrite (NO_2_^−^), nitric oxide (NO), nitrous oxide (N_2_O) and finally dinitrogen gas (N_2_) [172]. This process allows the bacteria to generate energy when oxygen is limited [172]. Enzymes such as nitrate reductase, nitrite reductase, nitric oxide reductase and nitrous oxide reductase catalyze each step [171]. The transcription factor Anr senses low oxygen and upregulates these genes. Oxygen gradients within biofilms generate metabolic heterogeneity, creating subpopulations of fast-growing, slow-growing and persister cells. These differences enhance tolerance to antibiotics and oxidative stress [171].

Quorum sensing and c-di-GMP signaling integrate nutrient sensing with biofilm formation and virulence factor production. QS regulates siderophore synthesis, secondary metabolites such as phenazines, and extracellular enzymes. Phenazines act as electron shuttles, facilitating energy generation in oxygen-limited niches. c-d-GMP coordinates motility repression with matrix production, linking metabolism to biofilm structure [164].

Overall, the ability to efficiently acquire nutrients and dynamically reprogram metabolism underpins *P. aeruginosa’s* survival, biofilm development, and resistance to host defenses and antibiotics.

### 3.7. Antibiotic Resistance as a Virulence-Associated Trait

Antibiotic resistance in *P. aeruginosa* is increasingly recognized not only as a survival mechanism but also as a trait that can enhance pathogenic potential [172]. While resistance itself does not directly cause tissue damage, it enables bacteria to persist in hostile host environments and maintain populations capable of expressing classical virulence factors, including toxins, proteases, and biofilm components [40]. Certain resistance mechanisms can also reshape QS networks, stress responses, and metabolic pathways. For instance, overexpression of the MexCD-OprJ multidrug efflux pump has been shown to extrude the PQS precursor HHQ, reducing intracellular PQS levels and attenuating QS-regulated virulence expression, thereby illustrating how resistance-associated systems can reprogram virulence circuitry [173]. The ability to withstand antibiotic pressure allows resistant strains to establish chronic infections, contributing to long-term pathogenic fitness and complicating treatment outcomes. Therefore, antibiotic resistance can be viewed as a virulence-associated trait, linking bacterial survival strategies with sustained pathogenicity [174].

**Figure 1:**
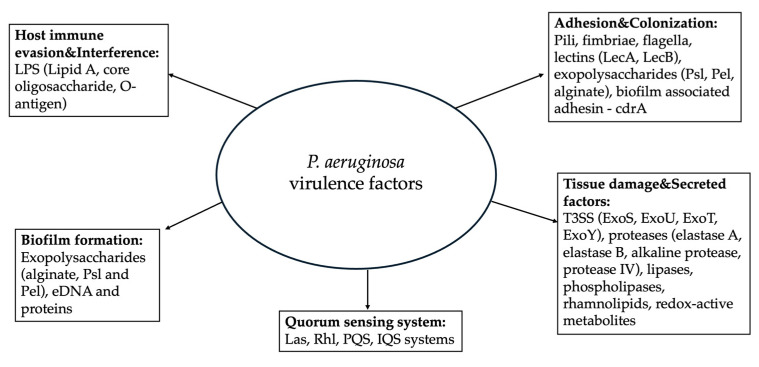
*P. aeruginosa* key virulence factors. Abbreviations: LPS, Lipopolysaccharide; T3SS, Type 3 secretion system; eDNA, extracellular DNA.

Figure 1, shown below, summarizes the key molecular mechanisms of *P. aeruginosa* described in the previous sections, highlighting how they collectively shape its pathogenic potential.

## 4. Strain-Specific Virulence and Clinical Phenotypes of *P. aeruginosa*

Different *P. aeruginosa* strains produce distinct clinical phenotypes. These differences arise from lineage-specific virulence repertoires, regulatory mutations and resistance backgrounds determine tissue tropism and pathogenic strategy [175].

Strains carrying the type III secretion effector exoU are reproducibly associated with fulminant cytotoxic disease and worse outcomes in ventilator-associated pneumonia and keratitis compared with exoS-positive strains, which instead favor intracellular survival and dissemination [94].

In chronic airway infections, loss-of-function mutations in the QS master regulator lasR are frequently selected. These variants typically display reduced acute virulence but enhanced biofilm formation, metabolic rewiring and long-term persistence [166].

Globally disseminated high-risk lineages, for example ST235 and ST111, combine transferable β-lactamases and overexpressed efflux systems (e.g., Mex pumps) with accessory virulence loci, explaining their association with nosocomial outbreaks, bloodstream infections and poor clinical outcomes [176].

A similar interplay between virulence regulation and niche adaption is observed in urinary tract infections, particularly catheter-associated UTI. Clinical isolates are often enriched for traits that promote surface colonization and show QS-regulated production of rhamnolipids and proteases that stabilize biofilms formed on catheter surfaces and increase antibiotic tolerance. Notably, some urinary isolates are also capable of invading and persisting intracellularly within bladder epithelial cells, undergoing transcriptional reprogramming that promotes antibiotic tolerance and recurrence in animal models [177].

## 5. Current Advances in Quorum Sensing Inhibition and Vaccine Development in *P. aeruginosa*

In recent years, advances in molecular microbiology have significantly improved our understanding of how *P. aeruginosa* causes disease. Its pathogenic potential is not driven by a single factor, but by a coordinated network of virulence determinants, including quorum sensing systems, the type III secretion system, biofilm-forming pathways, and a wide range of secreted toxins and enzymes. This knowledge has shifted the therapeutic perspective: instead of focusing exclusively on killing the bacterium, researchers are exploring ways to interfere with the specific mechanisms that enable it to damage host tissues, evade immunity, and persist in chronic infections. Targeting these processes directly may weaken the pathogenic impact while reducing selective pressure that accelerates antibiotic resistance.

Quorum sensing inhibition has emerged as one of the most intensively studied virulence-targeted strategies against *P. aeruginosa*. By disrupting the Las and Rhl regulatory systems, quorum sensing inhibitors (QSIs) aim to attenuate pathogenicity without directly inhibiting bacterial growth. A landmark study by O’Loughlin et al. designed and evaluated synthetic small molecules that interfere with QS in *P. aeruginosa* by targeting the key QS receptors LasR and RhlR [178]. Among the tested compounds, meta-bromo-thiolactone (mBTL) was identified as the most effective compound at blocking QS dependent signaling. Treatment with mBTL reduced production of the QS-controlled virulence factor pyocyanin and significantly impaired biofilm formation. Importantly, mBTL protected *Caenorhabditis elegans* as well as human lung epithelial cells from QS-mediated killing, demonstrating that inhibition of QS can attenuate virulence in both simple and mammalian models [178].

Recent studies continue to refine and expand this strategy. For example, a 2025 investigation identified fungal-derived QSIs that suppress QS-regulated virulence factor production and enhance antibiotic activity against resistant *P. aeruginosa* strains, highlighting ongoing efforts to discover chemically diverse and clinically viable QSI candidates [179]. Despite encouraging preclinical data, QSIs have not yet reached clinical approval. This is largely due to complexity and redundancy of QS regulatory networks, variability among clinical isolates and the need to demonstrate consistent in vivo efficacy, safety, and pharmacokinetic performance in humans [180].

In addition to antimicrobial and antivirulence strategies, vaccine development against *P. aeruginosa* has emerged as a promising preventive and adjunct therapeutic strategy aimed at reducing reliance on conventional antibiotics. Various vaccine platforms have been investigated to achieve this goal, including polysaccharide-based, subunit proteins, conjugate, DNA/mRNA and multicomponent formulations. Early vaccines targeted LPS and O-polysaccharides to induce opsonic antibodies, but the extensive serotype heterogeneity and limited immunogenicity have constrained their clinical success, and no licensed vaccine exists despite decades of research efforts [181,182]. Subunit vaccines incorporating conserved outer membrane proteins such as OprF, OprI and T3SS components have shown immunogenicity in preclinical models. However, high antigenic diversity, immune evasion mechanisms, and inconsistent protection across strains remain critical obstacles [181,183]. More recent strategies include multivalent protein subunit and nanoparticle-based vaccines designed to broaden antigenic coverage, as well as DNA/mRNA-based platforms leveraging novel delivery systems to elicit robust humoral and cellular responses [184]. Despite these advances, challenges such as selecting optimal antigens that provide broad cross-serotype protection, overcoming biofilm-associated immune suppression, and translating promising preclinical findings into human efficacy still impede clinical development of an effective *P. aeruginosa* vaccine [185].

## 6. Conclusions

*P. aeruginosa* exhibits a remarkable capacity to orchestrate a diverse repertoire of virulence factors—including adhesins, secreted toxins, biofilm formation and nutrient acquisition—through tightly regulated networks such as quorum sensing and c-di-GMP signaling. This coordinated adaptability allows the bacterium to persist under hostile host conditions, evade immune defenses, and resist antibiotic treatment.

*P. aeruginosa* virulence is not defined by a single toxin or factor, but by the bacterium’s ability to integrate classical virulence traits with adaptive survival strategies, enabling persistence, chronic infection, and resistance to host defenses and treatment.

Consequently, the extensive diversity and regulatory integration of *P. aeruginosa* virulence determinants provide multiple promising targets for the development of novel therapeutic strategies, including approaches aimed at disrupting signaling pathways, biofilm formation, and adaptive survival mechanisms rather than relying solely on conventional antibiotics.

## Data Availability

No new data were created or analysed in this study. Data sharing is not applicable to this article.

## Abbreviations

The following abbreviations are used in this manuscript:

WHO	World Health Organization
ADPRT	ADP-ribosyltransferase
CF	Cystic fibrosis
COPD	Chronic obstructive pulmonary disease
ECM	Extracellular matrix
eDNA	extracellular DNA
EPS	Exopolysaccharides
GAP	GTPase-activating domain
LPS	Lipopolysaccharides
MDR	Multidrug resistant
NET	Neutrophil extracellular trap
PC	phosphatidylcholine
PlcH	The hemolytic phospholipase C
PlcN	The non-hemolytic phospholipase C
PLCs	Phospholipases C
PldD	Phospholipase D
ROS	Reactive oxygen species
SM	Sphingomyelin
T3SS	Type III secretion system
TLR5	Toll-like receptor—5
QSI	Quorum sensing inhibitor
